# Intracellular expression of toll-like receptor 4 in neuroblastoma cells and their unresponsiveness to lipopolysaccharide

**DOI:** 10.1186/1471-2407-6-281

**Published:** 2006-12-08

**Authors:** Ferdaus Hassan, Shamima Islam, Gantsetseg Tumurkhuu, Yoshikazu Naiki, Naoki Koide, Isamu Mori, Tomoaki Yoshida, Takashi Yokochi

**Affiliations:** 1 Department of Microbiology and Immunology, Aichi Medical University School of Medicine, Nagakute, Aichi 480-1195, Japan

## Abstract

**Background:**

Recently it has been reported that, toll-like receptors (TLRs) are expressed on a series of tumor cells, such as colon cancer, breast cancer, prostate cancer, melanoma and lung cancer. Although some cancer cells like melanoma cells are known to respond to lipopolysaccharide (LPS) via TLR4, not all cancer cells are positive for TLR4. There is little information on the expression and function of TLR4 in neuroblastoma cells. In this study, we investigated the expression of TLR4 in human neuroblastoma NB-1 cell line.

**Methods:**

Expression and localization of TLR4 were detected by reverse transcription-polymerase chain reaction (RT-PCR) and flow cytometric analysis, respectively. Activation of nuclear factor (NF)-κB by LPS was detected by degradation of IκB-α and NF-κB luciferase assay. Activation and expression of mitogen-activated protein (MAP) kinase and interferon regulatory factor (IRF)-3 was detected by immunoblot analysis.

**Results:**

Human NB-1 neuroblastoma cells expressed intracellular form of TLR4, but not the cell surface form. Further, NB-1 cells express CD14, MD2 and MyD88, which are required for LPS response. However, LPS did not significantly induce NF-κB activation in NB-1 cells although it slightly degraded IκB-α. NB-1 cells expressed no IRF-3, which plays a pivotal role on the MyD88-independent pathway of LPS signaling. Collectively, NB-1 cells are capable to avoid their response to LPS.

**Conclusion:**

Although human NB-1 neuroblastoma cells possessed all the molecules required for LPS response, they did not respond to LPS. It might be responsible for intracellular expression of TLR4 or lack of IRF-3.

## Background

Recently it has been reported that, toll-like receptors (TLRs) are expressed on a series of tumor cells, such as colon cancer, breast cancer, prostate cancer, melanoma and lung cancer [1]. In particular, melanoma cells are reported to express TLR-4, respond to lipopolysaccharide (LPS) and produce interleukin (IL)-8 [2]. Thus, a direct link between tumor cell activation and LPS might be suggested because some tumor cells constitutively express TLR-4 [1]. However, not all tumor cells are positive for TLR-4 and it seems to depend on the tissue from which tumor cells originated.

Among neural cells, microglia cells are resident immune cells of the brain and express almost all TLRs [3-6]. Apart from microglia, astrocytes also differentially express TLRs with a robust expression of TLR-2 and a low level of expression of TLR-1, 4, 5 and 9 [5]. It is unclear whether neuroblasts express TLR-4 or not. Considering that neuroblastoma cells do not express TLR-4 [2], neuroblasts are unlikely to express TLR-4. In the present study, we examined two neuroblastoma cell lines to confirm the lack of TLR-4 expression in neuroblatoma cells. Surprisingly, we found that NB-1 neuroblastoma cells expressed intracellular TLR-4 but did not respond to LPS. Here, we discuss a possible mechanism of the unresponsiveness of intracellular TLR-4-expressing NB-1 neuroblastoma cells to LPS.

## Methods

### Materials

LPS from *Esherichia coli *O55:B5 was obtained from Sigma Chemicals, St. Louis, MO, USA. Phycoerythrin (PE)-conjugated anti-human TLR-4 antibody was obtained from eBioscience, San Diego, CA, USA. Antibody to human TLR-4 was purchased from Santa-Cruz biotechnology, Santa-Cruz, CA, USA. Antibody to phosphorylated interferon regulatory factor (IRF)-3 was from Upstate, Lake placid, New York, USA. Antibody to p38, c-Jun N-terminal kinase (JNK1/2), IκB, IRF-3, and phosphorylated form of p38 and JNK1/2 were purchased from Cell Signaling Technology, Beverly, MA, USA. Human interferon (IFN)-γ was purchased from Calbiochem, San Diego, CA, USA. Human IL-1β was purchased from R & D Inc, Concord, MA, USA.

### Cell culture

Human neuroblastoma cell lines, NB-1 and SK-N-SH, and human monocytic cell line, U-937, were obtained from Riken Cell bank (Tsukuba, Japan). NB-1 and U-937 cells were maintained in RPMI containing 5% heat inactivated fetal calf serum (GIBCO-BRL, Gaithersburg, MD, USA) and SK-N-SH cells was maintained in MEM with 10% heat-inactivated fetal calf serum. Cells were cultured in the presence of antibiotics at 37°C under 5% CO2.

### Laser flow cytometric analysis

NB-1 cells were seeded (1 × 10^6^/ml) in 35 mm dish for overnight, trypsinized and washed with phosphate-buffed saline (PBS). To detect cell surface expression of TLR4, the cells were incubated with PE-conjugated anti-TLR4 antibody for 20 min in dark on ice, washed and resuspended in PBS. To detect intracellular expression of TLR4, the cells were fixed with a fixation buffer (eBioscience) in dark and treated with a permeabilization buffer (eBioscience) according to the instruction of the manufacturer. The cells were stained with PE-conjugated anti-TLR-4 antibody. Cell surface or intracellular TLR-4-positive cells were analyzed by a fluorescence activated cells sorter (BD FACS Calibur, San Jose, CA, USA). PE-conjugated anti-rat isotype IgG was used as a negative control for the experiments.

### Reverse transcription-polymerase chain reaction (RT-PCR)

RT-PCR was performed as described previously [7]. Briefly, NB-1 and SK-N-SH cells were seeded (1 × 10^6^/ml) in 35 mm dish for overnight, trypsinized and washed with PBS. RNA was extracted from the cells with RNeasy mini kit (Qiagen, Valencia, CA, USA). Semi-quantative RT-PCR was carried out by using access quick RT-PCR system (Promega, Madison, WI, USA). Primer Sequence for TLR4, Forward 5 TGGATACGTTTCCTTATAAG 3 and Reverse 5 GAAATGGAGGCACCCCTTC 3 [8], CD14, forward 5 CTGCAACTTCTCCGAACCTC 3 and reverse 5 TAGGTCCTCGAGCGTCAGTT 5, MD2, forward 5 TTCCACCCTGTTTTCTTCCA 3 and reverse 5 AATCGTCATCAGATCCTCGC 3, MyD88, forward 5 GCACATGGGCACATACAGAC 3 and reverse 5 TGGGTCCTTTCCAGAGTTTG 3 [9]. GAPDH for TLR4 and G3PDH for CD14, MD2, MyD88 was used as an equal loading control. Optimized reverse transcription and PCR conditions were as follows, TLR4 (48°C for 45 min followed by 95°C for 2 min and 30 cycles at 95°C 30 sec, 56°C for 30 sec, 72°C for 60 sec), CD14, MD2 and MyD88 (48°C for 45 min followed by 95°C for 2 min and 28 cycles at 95°C 30 sec, 60°C 30 sec, 72°C for 1 min). For each experiment RNA extracted from human monocyte U937 was used as a positive control for TLR4, CD14, MD2 and MyD88. PCR products were analyzed by electrophoresis on 2% agarose gel. The gels were stained with CYBR safe DNA gel stain (Molecular probe, Eugene, OR, USA) and visualized under an ultraviolet transilluminator. The 100 bp DNA size marker (Invitrogen, Carlsbad, CA, USA) was also run to determine approximate size of the product.

### Immunoblotting

Immunoblotting was performed as described previously [7]. To differentiate monocyte to machrophages, U937 cells were treated with PMA for 24 hours, washed and incubated for another 24 hours before treated with LPS as indicated below. NB-1 cells were directly seeded in 35 mm culture dish, treated with LPS (100 ng/ml) for various time. The cell lysates were extracted by lysis buffer containing 0.5 M Tris-HCL, 4% SDS and 2 mercaptoethanol, and boiled at 80°C for 5 min. The protein concentration of each sample was determined by BCA protein assay reagent (Pierce, Rockford, IL, USA). An equal amount of protein (40 μg) was analyzed by SDS-polyacrylamide gel electrophoresis under reducing conditions and transferred to a membrane filter. The membranes were treated with an appropriately diluted antibody for overnight. The immune complexes were detected with a 1:5000 dilution of horseradish peroxidase-conjugated protein G for 1 h and the bands were visualized with a chemiluminesent reagent (Pierce, Rockford, IL). The chemiluminescence was analyzed by a light capture system (AE6955, Atto Corp., Tokyo, Japan) with CS grab, version 1.0. Densimetric analysis of each band was analyzed with CS analyzer software, version 1.0 (Atto Corp., Tokyo, Japan).

### Luciferase reporter gene assay for NF-κB activation

The luciferase reporter gene assay was performed as described previously [10]. Briefly, NB-1 cells (3 × 10^5^/ml) were plated in a 35 mm plastic dish. On the following day, the cells were transfected with 0.5 mg/ml of pNF-κB-TA-luc luciferase reporter gene (Invitrogen, Carlsbad, CA, USA) and 0.05 mg of pRL-TK plasmid (Promega, Madison, WI, USA) by lipofectamine 2000 transfection reagent (Gibco-BRL). The transfected cells were incubated for 48 h, stimulated with LPS (100 ng/ml) and IL-1β (100 ng/ml) for 6 h, lysed with a lysis reagent from Promega, and the luciferase activity was determined by the dual luciferase assay kit (Promega). To confirm the activation of NF-κB, we used another plasmid pNiFty2-Luc from Invivogen, San-Diego, CA, USA.

### Statistical analysis

Statistical significance was determined by Student's t test. Experimental result is expressed as the mean of triplicates ± SD in at least 3 independent experiments.

## Results

### NB-1 cells express TLR4

**Figure 1:**
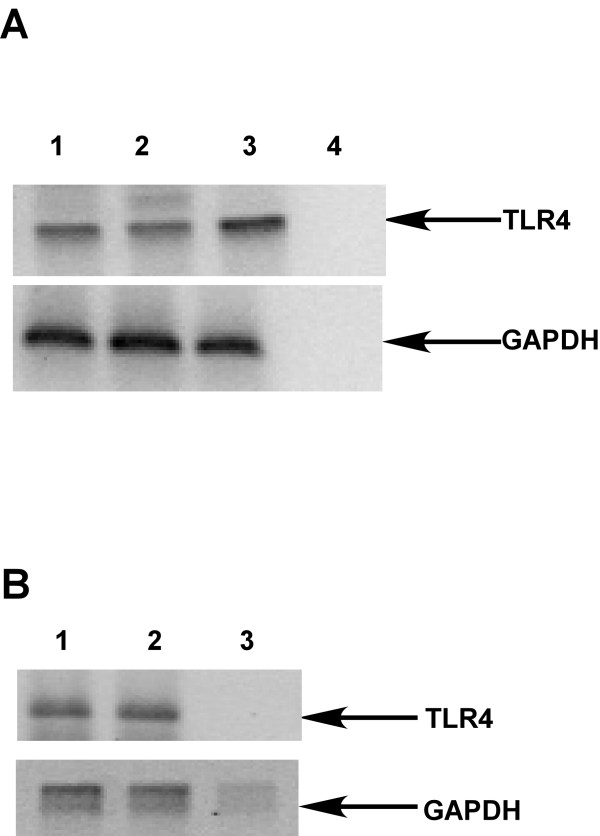
Expression of TLR4 mRNA in NB-1 and SK-N-SH neuroblastoma cells. A, NB-1 cells were incubated with LPS at 100 ng/ml for 4 h. Untreated NB-1 (1), LPS-treated NB-1 cells (2) and untreated U937 cells (3) were analyzed by RT-PCR. B, SK-N-SH cells (l) and U937 cells (2) were analyzed by RT-PCR. GAPDH mRNA was used as an equal loading control. Lane 4 and Lane 3 of each figure is used as a negative control respectively i.e RNA without reverse transcriptase enzyme (R.T). Data is the representative of at least three independent experiments.

By using RT-PCR, we investigated whether human neuroblastoma cell line NB-1 expresses TLR4 or not. NB-1 cells were also treated with or without LPS (100 ng/ml) for 4 hr. The expression of TLR4 was examined with RT-PCR using extacted RNA. As shown in Fig. 1, NB-1 cells definitely express TLR4 mRNA (Fig 1A, lane 2). LPS treatment did not affect the expression of TLR4 (Fig 1A, Lane 3). To exclude the exceptional expression of TLR4 in NB-1 cells, we examined TLR4 expression in another human neuroblastoma cell line SK-N-SH. RT-PCR analysis demonstrated that SK-N-SH cell line as well as NB-1 cell line expressed TLR4 mRNA (Fig. 1B), suggesting that two neuroblastoma cell lines might commonly express TLR4.

### Intracellular but not cell surface expression of TLR4 in NB-1 cells

**Figure 2:**
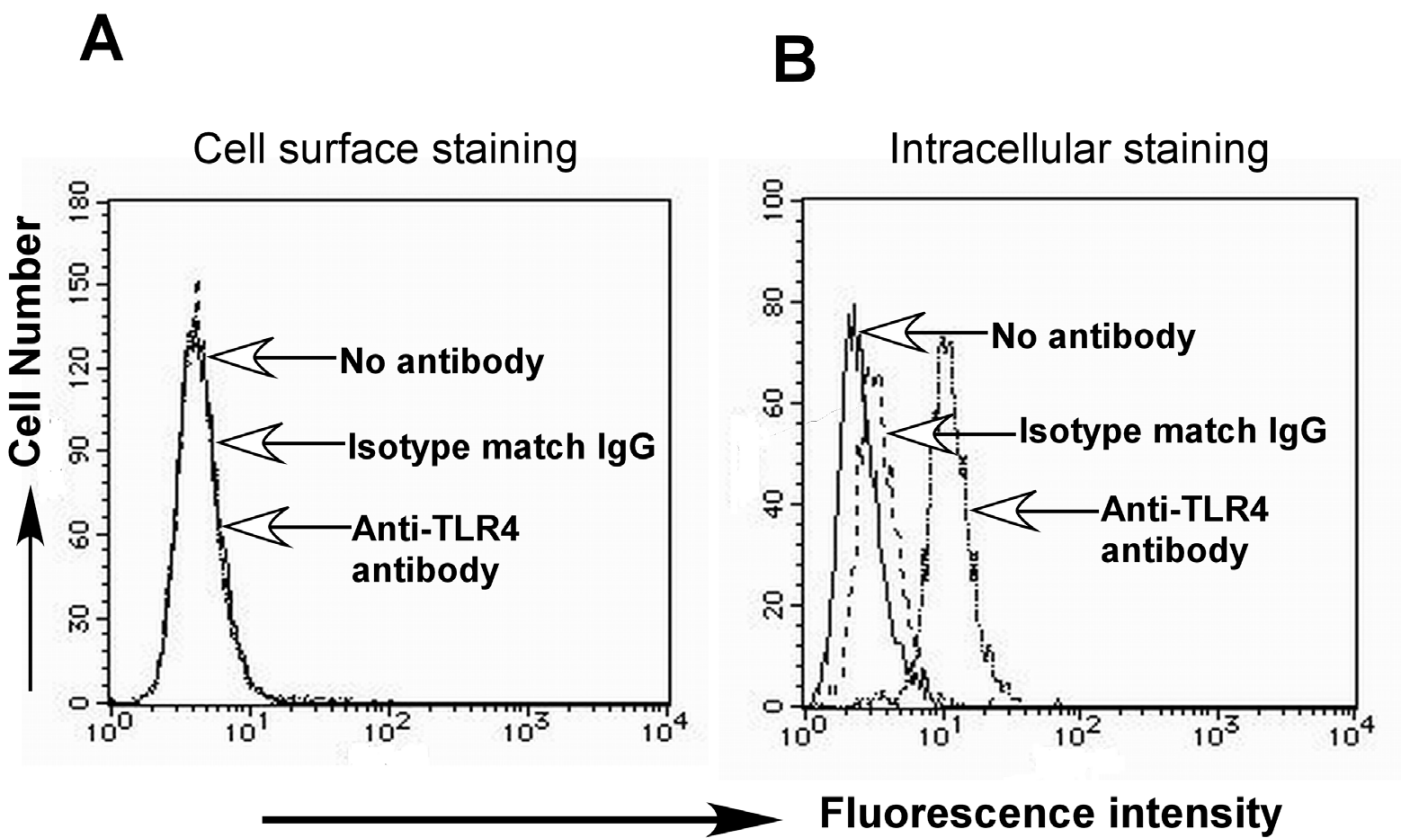
Intracellular localization of TLR4 in NB-1 cells. A, NB-1 cells were stained with no antibody, PE-conjugated isotype match IgG or PE-conjugated anti-TLR4 antibody. B, Permeabilized NB-1 cells were stained with the same protocol. The fluorescent histogram was analyzed by a laser flow cytometer. Data is the representative of at least three independent experiments.

In mammalian cells, TLRs are expressed either on cell surface or in the cytoplasm i.e golgi apparatus [11]. For example, TLR1, TLR2 and TLR4 are located on cell surface and are recruited to phagosomes after activation by their respective ligands whereas TLR3, TLR7 and TLR9 which recognize nucleic acid-like structures are located in subcellular compartment [12-16]. First, we investigated the cell surface expression of TLR4 on NB-1 cells with flow cytometric analysis. NB-1 cells were stained with either PE-conjugated anti-human TLR4 antibody or PE-conjugated isotype matched IgG. Positively stained cells were not detected in NB-1 cells (Fig 2A), suggesting no cell surface expression of TLR4. In addition, U937 cells as positive control were strongly positive for TLR4. Second, we examined the intracellular expression of TLR4 using permeabilized NB-1 cells because RT-PCR analysis demonstrated the presence of TLR4 mRNA. The histogram of permeabilized NB-1 cells stained by anti-TLR4 antibody shifted to the right side (Fig. 2B), suggesting the intracellular localization of TLR4.

### NB-1 cells express CD14, MD2 and MyD88 which participate in TLR4 signaling pathway

**Figure 3:**
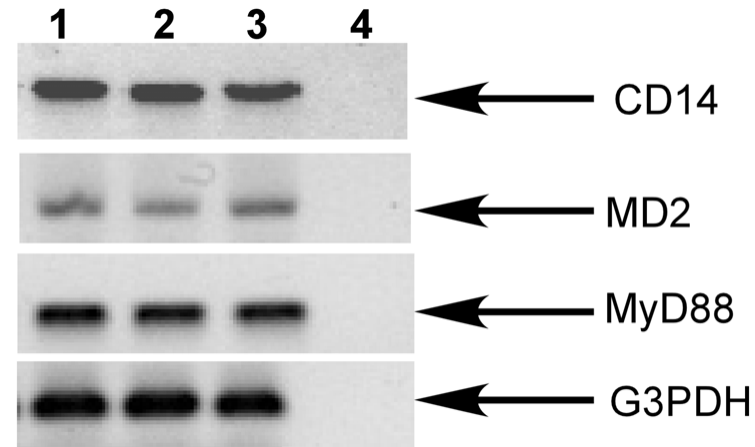
Expression of CD14, MD2 and MyD88 in NB-1 cells. NB-1 cells were incubated with or without LPS at 100 ng/ml for 4 h. RNA was extracted and used for RT-PCR. Lane 1, untreated NB-1 cells; lane 2, LPS-treated NB-1 cells; lane 3, untreated U937 cells. Lane 4 is used as a negative control i.e RNA without reverse transcriptase enzyme (R.T). Data is the representative of at least three independent experiments.

There are several key molecules leading to TLR4-dependent cellular activation by LPS. Besides TLR4, a series of molecules, such as CD14, MD2 and MyD88 are essential for LPS response [17,18]. First, we studied the expression of CD14, MD2 and MyD88 mRNA on NB-1 cells. RNA was extracted from NB-1 cells treated with or without LPS (100 ng/ml) for 4 hr and used to RT-PCR for CD14, MD2 and MyD88. RT-PCR analysis demonstrated that mRNAs of the three molecules are expressed in NB-1 cells (Fig. 3). In addition, LPS treatment did not alter their expression in NB-1 cells. Human monocytic cell line U937 as a positive control was also positive for CD14, MD2 and MyD88 mRNA. Laser flow cytometric analysis demonstrated cell surface expression of CD14 in NB-1 cells (Data not shown).

### LPS partially degrades IκB-α

**Figure 4:**
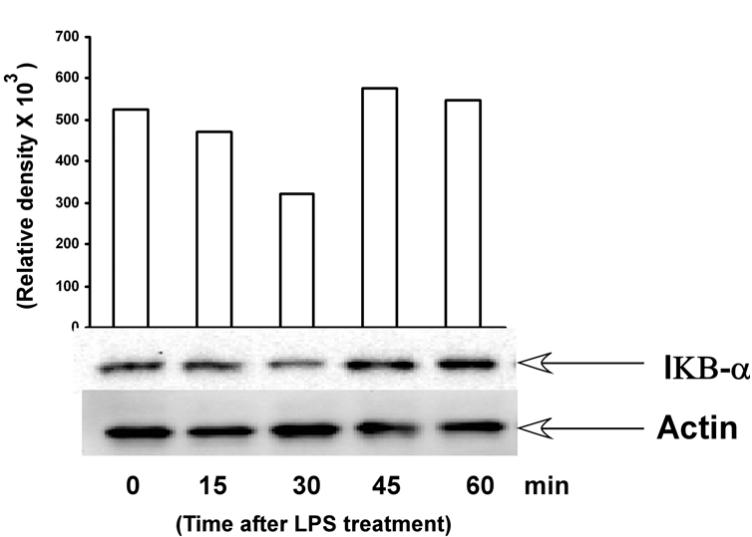
Effect of LPS on IκB-α degradation in NB-1 cells. NB cells were cultured with LPS at 100 ng/ml for various time and cell extracts were analyzed with immunoblotting using anti-IκB-α antibody. Densitometric analysis of each IκB-α band was performed by a CS analyzer (top). A typical experimental result of three independent experiments is shown.

It has been well documented that NF-κB associated with IκB resides in cytoplasm as an inactive form. On the process of NF-κB activation, IκBα is rapidly phosphorylated and degraded and then free NF-κB is translocated to nucleus [19]. In order to investigate whether LPS is able to activate NF-κB via intracellular TLR4, we examined degradation of IκB-α in NB-1 cells treated with LPS (100 ng/ml) for various time. As shown in Fig. 4, the expression of IκB-α partially decreased up to 30 min after LPS treatment and it returned to the original state 45 and 60 min after LPS treatment. The possibility that a small amount of contaminants in the LPS preparation might induce the partial activation still remained.

### LPS does not activate NF-κB in NB-1 cells

**Figure 5:**
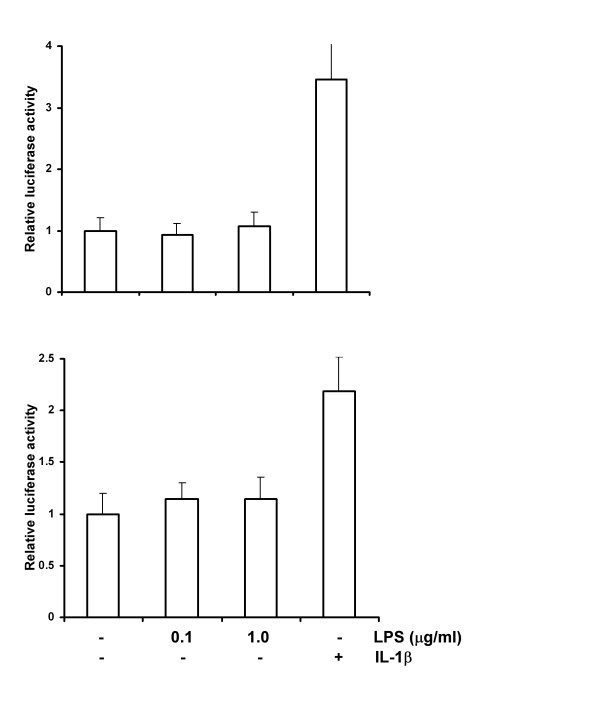
Effect of LPS on NF-κB activation in NB-1 cells. Transfected NB-1 cells were treated with LPS at various concentrations for 6 h. The NF-κB activity was expressed as relative luciferase activity by fold increase. IL-1β (0.1 μg/ml) was used as a positive inducer of NF-κB. A typical experimental result of three independent experiments is shown.

Based on partial degradation of IκB-α upon LPS treatment, we studied the activation of NF-κB by NF-κB-dependent luciferase assay. NB-1 cells transfected with the reporter gene were treated with LPS at various concentrations for 6 h. Human IL-1β as a positive inducer [20] was used at 100 ng/ml for 6 hr and the NF-κB activity was determined with a relative luciferase activity. LPS at 5 μg/ml failed to activate NF-κB activity in NB-1 cells whereas IL-1β markedly enhanced it (Fig. 5). Further, we tried another NF-κB-inducible reporter plasmid pNiFty2-Luc to detect NF-κB activation. However, LPS failed to activate NF-κB although IL-1β activated it significantly. Further, treatment with LPS for 24 h did not induce NF-kB-dependent TNF-α production in NB-1 cells (data not shown).

### NB-1 cells do not express IRF-3

**Figure 6:**
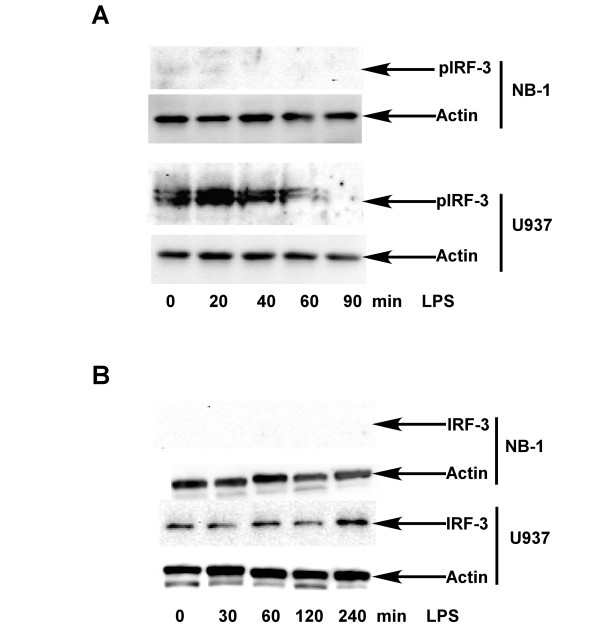
Effect of LPS on IRF-3 activation in NB-1 cells. NB-1 cells were cultured with LPS at 100 ng/ml for various time as indicated. Cell extracts were analyzed with immunoblotting using an antibody to phosphorylated form of IRF-3 (pIRF-3) (A) or total IRF-3 (B). U937 cells were used as a positive control. Equal loading is shown using anti-actin antibody. A typical experimental result of three independent experiments is shown.

Among all TLRs, only TLR3 and TLR4 can induce cellular activation in both MyD88 dependent and independent pathway [21]. In the MyD88-independent pathway, LPS treatment leads to activation of transcription factor IRF-3, followed by production of IFN-β. Subsequently, IFN-β activates STAT1, leading to the induction of several IFN-inducible genes [22-24]. We examined a possibility that NB-1 cells might have a defect in MyD88-independent pathway, especially IRF-3. Immunoblotting analysis demonstrated that LPS induces phosphorylation of IRF-3 in U937 but not in NB-1 cells. (Fig. 6A). Further, we examined the expression of IRF-3 in NB-1 cells and U937 cells. Immunoblotting analysis demonstrated that NB-1 cells did not express IRF-3 (Fig. 6B) although it was definitely expressed in U937 cells.

### LPS does not activate MAP kinases in NB-1 cells

**Figure 7:**
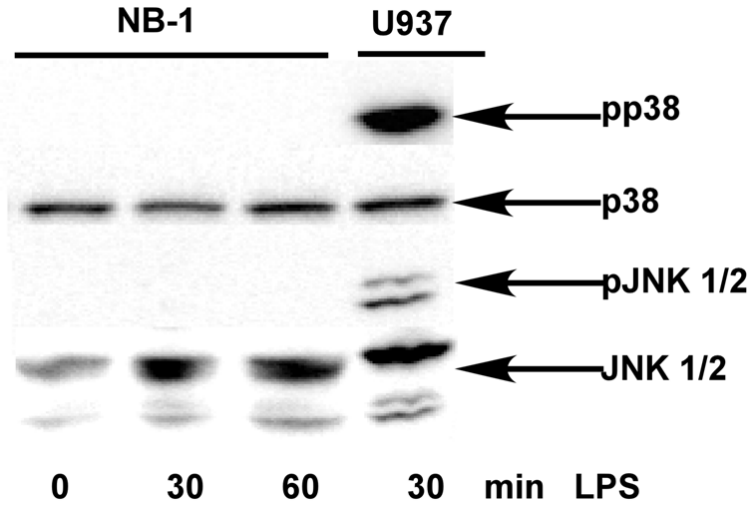
Effect of LPS on MAPK in NB-1 cells. NB-1 cells were cultured with LPS at 100 ng/ml for 30 and 60 min. The cell extracts were analyzed with immunoblotting using an antibody to p38, JNK 1/2 or the phosporylated form of p38 (pp38) and JNK1/2 (pJNK1/2). U937 was used as a positive control. A typical experimental result of three independent experiments is shown.

LPS is known to activate JNK1/2 and p38 MAP kinases through TAK1, a downstream molecule of TLR4-mediated signaling pathway [25]. We examined the activation of JNK1/2 and p38 MAP kinases in LPS-treated NB-1 cells. LPS did not activate JNK1/2 and p38 MAP kinases in NB-1 cells until 60 min (Fig. 7) whereas LPS activated them at 30 min in U937 cells. In addition, the treatment with LPS for 2 h also failed to activate p38 and JNK1/2 in NB-1 cells (data not shown).

## Discussion

In the present study we have demonstrated that NB-1 and SK-N-SH neuroblastoma cells express intracellular TLR-4. On the other hand, SHSY-5Y neuroblastoma cells are reported to express no TLR4 at mRNA level [2]. Therefore, there seemed to be a difference in TLR4 expression among various neuroblastoma cell lines. Although TLR4 is usually expressed on cell surface, there are a number of reports on intracellular expression of TLR4 in various cell types and cell lines, such as human placenta, coronary artery endothelial cells, dendritic cells, pulmonary epithelial cells, and intestinal epithelial cells m-ICc12 [11,26-29]. Further, intracellular TLR4 is reported to responds to LPS [11,26-29]. However, NB-1 cells expressing intracellular TLR4 did not respond to LPS.

NB-1 neuroblastoma cells express TLR4, MD2, CD14 and MyD88, which are required for LPS signaling. LPS slightly degraded IκB-α but did not significantly lead to NF-κB activation in NB-1 cells. On the other hand, NF-κB signaling acts normally because IL-1β induced the activation of NF-κB in those cells. Therefore, intracellular TLR4 expression might be involved in the unresponsiveness of NB-1 cells to LPS. LPS may not be internalized in NB-1 cells. Otherwise, internalized LPS may undergo deacylation by lysomal enzymes and become inactive before reaching Golgi apparatus where TLR4 reside. Further, we can not exclude a possibility that the LPS unresponsiveness may be due to deficiency of some molecule(s) required for LPS signaling. Interestingly, we found no expression of IRF-3 in NB-1 cells. Kawai *et al*. reported that IRF-3 plays a central role in MyD88-independent pathway [30]. Therefore, the MyD88-independent pathway of LPS signaling may not occur in NB-1 cells. The impairment of MyD88-independent pathway with defected IFR-3 might be related to the unresponsiveness of NB-1 cells to LPS.

Thieblemont *et al*. demonstrated that LPS is transported to Golgi apparatus [31]. Further, Hornef *et al*. reported that TLR4 resides in the Golgi apparatus and cellular trafficking of LPS to Golgi apparatus is important for innate immune response [11]. On the other hand, there are several contradictory reports on it. Latz *et al*. reported that LPS traffics to the Golgi apparatus with TLR4-CD14-MD2 complex but does not initiate the signal transduction pathway and that TLR4 must reside on cell surface to trigger the signaling pathway, [32]. Even when LPS was artificially introduced into the cytoplasm, human coronary epithelial cells were failed to activate due to its intracellular localization of TLR4 [33]. Our present study supports a possibility that LPS might fail in triggering NF-κB activation in NB-1 cells through intracellular TLR4 expression or impaired MyD88-independent signaling.

## Conclusion

In this report we for the first time demonstrate that neuroblastoma cells express TLR4 at the cytoplasm but not at the cell surface. We further showed that, neuroblastoma cell line NB-1 does not respond to LPS. We conclude that unresponsiveness to LPS might be due to intracellular localization of TLR4 or lack of IRF-3 expression. Further studies must be necessary to confirm the expression and role of TLR4 in neuroblastoma cells and neuroblasts.

## Competing interests

The author(s) declare that they have no competing interests.

## Authors' contributions

FH carried out the study, participated to design the study, wrote the first draft of the manuscript, SI and GT carried out the PCR analysis, YN and NK participated to design the study and helped to write the manuscript, IM was involved to revise the manuscript, T Yoshida participated for the final design and writing the manuscript, T Yokochi designed and supervised the whole study and wrote the final draft of the manuscript. All authors read and approved the final manuscript

## Pre-publication history

The pre-publication history for this paper can be accessed here:
